# IL-13Rα2 Status Predicts GB-13 (IL13.E13K-PE4E) Efficacy in High-Grade Glioma

**DOI:** 10.3390/pharmaceutics14050922

**Published:** 2022-04-24

**Authors:** Julian S. Rechberger, Kendra A. Porath, Liang Zhang, Cody L. Nesvick, Randy S. Schrecengost, Jann N. Sarkaria, David J. Daniels

**Affiliations:** 1 Department of Neurologic Surgery, Mayo Clinic, Rochester, MN 55905, USA; rechberger.julian@mayo.edu (J.S.R.); zhang.liang@mayo.edu (L.Z.); nesvick.cody@mayo.edu (C.L.N.); 2 Department of Molecular Pharmacology and Experimental Therapeutics, Mayo Clinic Graduate School of Biomedical Sciences, Rochester, MN 55905, USA; 3 Department of Radiation Oncology, Mayo Clinic, Rochester, MN 55905, USA; porath.kendra@mayo.edu (K.A.P.); sarkaria.jann@mayo.edu (J.N.S.); 4 Targepeutics, Inc., Hershey, PA 17033, USA; randys@targepeutics.com

**Keywords:** high-grade glioma, diffuse midline glioma, glioblastoma, IL-13Rα2, IL-13, immunotoxin, targeted therapy, GB-13, IL13.E13K-PE4E, receptor expression

## Abstract

High-grade gliomas (HGG) are devastating diseases in children and adults. In the pediatric population, diffuse midline gliomas (DMG) harboring H3K27 alterations are the most aggressive primary malignant brain tumors. With no effective therapies available, children typically succumb to disease within one year of diagnosis. In adults, glioblastoma (GBM) remains largely intractable, with a median survival of approximately 14 months despite standard clinical care of radiation and temozolomide. Therefore, effective therapies for these tumors remain one of the most urgent and unmet needs in modern medicine. Interleukin 13 receptor subunit alpha 2 (IL-13Rα2) is a cell-surface transmembrane protein upregulated in many HGGs, including DMG and adult GBM, posing a potentially promising therapeutic target for these tumors. In this study, we investigated the pharmacological effects of GB-13 (also known as IL13.E13K-PE4E), a novel peptide–toxin conjugate that contains a targeting moiety designed to bind IL-13Rα2 with high specificity and a point-mutant cytotoxic domain derived from Pseudomonas exotoxin A. Glioma cell lines demonstrated a spectrum of IL-13Rα2 expression at both the transcript and protein level. Anti-tumor effects of GB-13 strongly correlated with IL-13Rα2 expression and were reflected in apoptosis induction and decreased cell proliferation in vitro. Direct intratumoral administration of GB-13 via convection-enhanced delivery (CED) significantly decreased tumor burden and resulted in prolonged survival in IL-13Rα2-upregulated orthotopic xenograft models of HGG. In summary, administration of GB-13 demonstrated a promising pharmacological response in HGG models both in vitro and in vivo in a manner strongly associated with IL-13Rα2 expression, underscoring the potential of this IL-13Rα2-targeted therapy in a subset of HGG with increased IL-13Rα2 levels.

## 1. Introduction

High-grade gliomas (HGG) encompass the majority of malignant tumors within the central nervous system (CNS), and with fewer than 25,000 new cases annually in the US, they are categorized as a rare disease [1]. H3K27-altered diffuse midline glioma (DMG), formerly known as diffuse intrinsic pontine glioma (DIPG), constitute a subset of HGG that predominantly occurs in children and makes up approximately half of the HGGs in this patient population [2,3]. These tumors are localized to the thalamus, brainstem and spinal cord and often lack contrast enhancement on magnetic resonance imaging (MRI), suggesting that they maintain a largely intact blood–brain barrier (BBB), an impediment to systemic drug delivery [4,5]. Despite the recent discovery of key molecular drivers of disease, clinical trials for DMG continue to fail, and palliative external beam radiotherapy remains the mainstay of therapy [6,7]. The prognosis for patients with DMG is dismal, with a median overall survival of 9 months and no long-term survivors [1,8]. In adults, glioblastoma (GBM) is the most prevalent and aggressive HGG subtype. The current standard treatment consists of maximal surgical debulking, radiotherapy, and concomitant and adjuvant temozolomide chemotherapy [9,10]. However, the diffuse, infiltrative nature and proclivity for recurrence render GBM largely intractable [1,2]. While clinical trials are underway utilizing a range of different therapeutic approaches, no treatment has demonstrated a benefit to standard of care by extending survival in this tumor in almost two decades, and the median survival is currently less than two years from diagnosis [11,12,13,14]. Consequently, the dire prognosis for patients with HGG and the lack of efficacious, targeted therapies for these tumors demand a novel approach to their treatment.

Interleukin 13 (IL-13) is an immune-regulatory cytokine implicated in both physiologic and tumoral microenvironments through effects on IL-13 receptor alpha 1 (IL-13Rα1) and IL-13 receptor alpha 2 (IL-13Rα2) receptors [15]. Normally, IL-13 binds to IL-13Rα1, with IL-4Rα providing stabilization to this interaction, thereby inducing formation of a receptor dimer [16]. The intracellular signaling axis downstream of IL-13Rα1/IL-4Rα promotes apoptotic signaling cascades via a caspase-dependent mechanism [17]. In contrast, IL-13Rα2 acts as a decoy receptor that directly binds IL-13 as a monomer with greater binding affinity than IL-13Rα1 [18]. When IL-13Rα2 is expressed on the surface of select cell types, IL-13 is sequestered away from IL-13Rα1, thus leading to escape from apoptotic cell death [19,20].

IL-13Rα2 is expressed almost exclusively on cancer cells and is a clinically validated target for biologic therapeutics. Malignant diseases with known IL-13Rα2 upregulation include but are not limited to HGG [16,21,22,23], malignant peripheral nerve sheath tumors [24], colon cancer [25], pancreatic cancer [26], ovarian cancer [27], and melanoma [28]. Recent studies suggest that overexpression of IL-13Rα2 is detected in up to 83% of malignant pediatric brain tumors, including DMG, and up to 78% of adult GBM [23,29,30,31]. Furthermore, IL-13Rα2 significantly correlates with poor prognosis in HGG [16]. Given the status of IL-13Rα2 as a promising therapeutic target in HGG, a number of preclinical studies and clinical trials have been conducted, demonstrating the safety and efficacy of chimeric antigen receptor (CAR)-engineered T cell-based therapies and recombinant immunotoxins directed against this target [32,33]. Previously, convection-enhanced delivery (CED) of cintredekin besudotox (IL13-PE38QQR), a wild-type IL-13 pseudomonal exotoxin (PE)-A conjugate that targets both IL-13Rα1 and IL-13Rα2, was evaluated in phase I and phase III clinical trials for DMG and GBM, respectively [33,34,35,36]. However, these studies were hampered by ill-defined inclusion criteria and did not consider the IL-13Rα2 expression status in tumors of enrolled subjects, which likely contributed to disappointing survival results [37].

The purpose of this study was to determine the impact of tumor-associated IL-13Rα2 levels on the therapeutic efficacy of IL-13Rα2-targeted therapy in HGG. We screened the novel recombinant chimeric immunotoxin GB-13 (IL13.E13K-PE4E) against a library of select HGG patient-derived cell lines. GB-13 consists of an N-terminal IL-13-targeting moiety with a single engineered point mutation, the C-terminus of which is linked to the N-terminus of a full-length PE molecule containing four point mutations. These modifications greatly enhance the selectivity and affinity for IL-13Ra2 and reduce toxicity to non-malignant cells [24,38,39]. The IL-13 moiety attaches to IL-13Ra2 at the cell surface of malignant target cells and facilitates the internalization of the toxin, which irreversibly disables eukaryotic elongation factor 2 (eEF2) by adenosine diphosphate (ADP)-ribosylation using oxidized nicotinamide adenine dinucleotide (NAD+), causing arrest of protein synthesis and eventual Bak- and caspase-mediated apoptosis [40,41,42]. Treatment with GB-13 resulted in dose-dependent killing of DMG and adult GBM cells in a manner strongly associated with IL-13Rα2 expression. Moreover, intratumoral administration of GB-13 via CED decreased tumor burden and prolonged survival in both DMG and adult GBM intracranial murine xenografts with high, but not low levels of IL-13Rα2. Our results illustrate a previously underappreciated role of IL-13Rα2 heterogeneity in HGG and outline the potential of IL-13Rα2-targeted therapies in a subset of these tumors.

## 2. Materials and Methods

### 2.1. Materials

The IL-13Rα2-targeted therapy used in this study was GB-13 (IL13.E13K-PE4E), which was a generous gift from Targepeutics Inc. (Hershey, PA, USA). GB-13 was dissolved in PBS and stored as 2.6 mg/mL stock at −80 °C. Human IL-13 recombinant protein (Cat # A42525; Thermo Fisher Scientific, Rockford, IL, USA) was obtained from Invitrogen (Thermo Fisher Scientific). IL-13 was dissolved in ddH_2_O per the manufacturer’s protocol and stored as 5 μg/mL stock at −80 °C.

### 2.2. Cell Lines and Culture

Informed consent and Institutional Review Board approval were obtained for all patient-derived cell lines. Details regarding cell lines can be found in Table S1. Early-passage HGG lines were used, and all cell lines were validated by short tandem repeat DNA fingerprinting annually and tested for Mycoplasma contamination every 3 months. Cell lines with the H3K27M mutation were validated for K27M-mutant histone expression using Western blot and Sanger sequencing every 3 months. All patient-derived tumor cell lines were maintained in cell-line-appropriate medium, the details of which are provided in Table S2. Cells cultured as neurospheres were passaged every 1–2 weeks. Cells cultured as adherent monolayers were passaged 1–2 times per week.

### 2.3. RNA Sequencing and Data Analysis

Total RNA was extracted from whole-cell lysates using the RNeasy Plus micro kit (Cat # 74034; QIAGEN, Germantown, MD, USA) according to the manufacturer’s instructions. For the purpose of screening a large library of cell lines, RNA-Seq studies were performed as single replicates. RNA library preparation and sequencing were performed by Novogene (Beijing, China). The NEBNext UltraTM RNA Library Prep Kit for Illumina sequencers (New England Biolabs, Ipswich, MA, USA) was used for library preparation, and cDNA libraries were subsequently size selected using AMPure XP magnetic beads (Beckman Coulter, Pasadena, CA, USA). Samples were sequenced on an NovaSeq 6000 sequencer (Illumina, San Diego, CA, USA) using either single- or paired-end sequencing, depending on the timeframe of sample availability and the sequencing technology available. Paired-end sequencing data on adult GBM cell lines were obtained from cBioPortal, a free web-based tool that contains RNA-Seq data on Mayo Clinic’s brain tumor patient-derived xenografts [43,44]. Generated FASTQ files underwent quality assessment using FASTQC (https://www.bioinformatics.babraham.ac.uk/projects/fastqc/, accessed on 22 September 2021). Trimmed reads were mapped to hg38 using STARv2.7.3a, and annotated gene counts were obtained using the –quantMode geneCounts function [45]. Transcripts (TPM) or reads per kilo base per million (RPKM) reads values were calculated using RSEM in a manner concordant with the single- or paired-end status of the library [46].

### 2.4. Immunoblotting

Patient-derived tumor cells for immunoblotting were lysed in Triton X-100 lysis buffer containing protease inhibitors and sonicated. Collected protein lysates were stored at −20 °C. Protein concentrations were determined using the Pierce BCA Protein Assay Kit (Cat #23227; Thermo Fisher Scientific). An amount of 15 μg of total protein was size fractioned by 12.5% SDS-PAGE. Electrophoresis-separated proteins were electrically transferred to a polyvinylidene difluoride (PVDF) membrane, washed in PBST buffer, and blocked in 2% fat-free milk for 1 h at room temperature, then incubated with primary antibodies at 4 °C overnight. Following primary antibody blotting, specific signal was detected with species-appropriate peroxidase-conjugated secondary antibody (Thermo Fisher Scientific) using SuperSignal West Pico PLUS Chemiluminescent Substrate (Cat #34580; Thermo Fisher Scientific) and imaged using an Azure 600 Western blot imaging system (Azure Biosystems, Dublin, CA, USA). Details regarding antibodies used for Western blots can be found in Table S3.

### 2.5. Cell Proliferation and Viability Assays

Cells in single-cell suspension were plated with culture medium in 96-well clear-bottom black microplates (Cat #3917; Corning Costar, Corning, NY, USA) at a density of 2500 cells per well for adult GBM cell lines (GBM6, GBM 10, GBM14, GBM 39, GBM43, and GBM 108) or 5000 cells per well for DMG cell lines (SU-DIPG XIII-P [47], SU-DIPG XVII [48], SF8628, SF8628-B23 (H3F3A K27M knockout of SF8628), and PED17) and cultured overnight at 37 °C with 5% CO_2_. The next day, cells were treated in triplicate with either vehicle (ddH_2_O or PBS) or serial dilutions of IL-13 (to final concentrations of 100, 50, 20, 10, 5, 1, and 0.5 ng/mL) or GB-13 (to final concentrations of 320, 100, 32, 10, 3.2, 1, 0.32, 0.1, 0.032, 0.01, 0.0032, and 0.001 ng/mL). Cells were incubated for 72 h and then assayed with CellTiter-Glo Luminescent Cell Viability Assay (Cat #G7570; Promega, Madison, WI, USA) according to the manufacturer’s recommendations. Luminescence was measured using an Infinite M200 PRO multimode microplate reader (Tecan Group, Männedorf, Switzerland), normalized to control wells (ddH_2_O or PBS only), and relative luminescence treatment was plotted as a function of drug concentration. The potency (50% inhibitory concentration, IC_50_) of each treatment was calculated by non-linear least-squares curve fitting using Prism 9 (GraphPad, San Diego, CA, USA).

### 2.6. Immunofluorescence

Cells were plated in single-cell suspensions at a density of 10,000 cells per well on 4 Chamber Cell Culture Slides (Cat # 50-114-9053; CELLTREAT Scientific Products, Pepperell, MA, USA) and cultured overnight at 37 °C with 5% CO_2_. After 24 h, cells were treated with either vehicle (PBS) or the IC_50_ concentration of GB-13, as determined by CellTiter-Glo Luminescent Cell Viability Assay (Promega). At specific timepoints (8, 24, 48, and 72 h), cells were then washed in PBS and fixed with 4% paraformaldehyde for 20 min. Cells were washed 3 times for 5 min each in PBS and incubated in 0.5% Triton X-100 in PBS for 5 min. To wash the coverslips of the permeabilization buffer, cells were incubated in PBS 3 times for 5 min each before blocking with 3% BSA in PBS-T (0.1% Tween 20) for 1 h at room temperature. Up to two different primary antibodies were then added in 1% BSA in PBST overnight at 4 °C. Dilution buffer was used in lieu of primary antibody for cell-specific negative controls. The next day, cells were washed 3 times for 5 min each in PBS-T. Cells were then incubated with Alexa Fluor-coupled secondary antibodies (Thermo Fisher Scientific) in 1% BSA in PBS-T for 1 h at room temperature in the dark. To test for cross-reactivity, one control per primary antibody condition was included by applying the other secondary antibody to the primary antibody. After three additional 5 min washes in PBS, chambers were removed and slides were rinsed thrice in ddH_2_O. Slides were mounted using ProLong Gold Antifade reagent with DAPI (Cat # P36935; Thermo Fisher Scientific) and stored at 37 °C until microscopy imaging. All slides were examined and images captured using a LSM 780 confocal laser scanning microscope (Carl Zeiss Microscopy, White Plains, NY, USA). Detailed information regarding antibodies used for immunofluorescence can be found in Table S3.

### 2.7. Patient-Derived Xenografts

All animal experiments were conducted in accordance with the NIH and IACUC guidelines for the use of animals in research and approved by the Mayo Clinic Institutional Committee for Animal Research. HGG cell lines (GBM6, PED17, and SU-DIPG XIII-P [47]) were transduced with a luciferase reporter system (eGFP/fLuc2) that allows bioluminescence readout of tumor volume [49]. Orthotopic tumor inoculation with cultured cells was performed as previously described [49,50]. Briefly, cells were placed in single-cell suspension, and 300,000 cells in 3 μL of sterile PBS were prepared for engraftment into each mouse. A 0.5 mm burr hole was created at the following coordinates: 1 mm posterior and 2 mm to the right of the bregma (GBM6) or 1 mm posterior to the lambdoid suture and 1 mm lateral to the mid-sagittal plane (PED17 and SU-DIPGXIII). Using a 26 gauge (51 mm, point style AS) syringe (Cat. #203185; Hamilton Company, Bonaduz, Switzerland), tumor cells were injected stereotactically at a constant flow rate of 0.5 μL/min into the cerebral hemisphere (GBM6) or pons (PED17 and SU-DIPGXIII) of 6- to 7-week-old female Hsd:Athymic Nude-Foxn1^nu^ mutant mice that were obtained from Charles River Laboratories (Wilmington, MA, USA). The injection depth was 4 mm for all groups. In vivo tumor engraftment and progression were monitored by bioluminescence imaging (BLI). Animals were dosed with an intraperitoneal injection of 10 mg/kg of Cycluc. After 10 min, mice were imaged under isoflurane anesthesia using an IVIS-200 Imaging System (Xenogen Corporation, Berkeley, CA, USA). Image analysis was performed using LivingImage 4.3 (PerkinElmer, Waltham, MA, USA) to quantitate total flux (number of photons per second) within a region of interest.

For brain-targeted drug delivery, animals were randomized to control (PBS) and treatment (GB-13) groups based on BLI signal to ensure equal distribution of tumor sizes at the beginning of the study (when BLI reached approximately 1,000,000 total log flux). Mice were placed under anesthesia with 100 mg/kg of ketamine and 10 mg/kg of xylazine. A 2 cm midline skin incision was made extending from behind the eyes to level of the ears. The previously established burr hole was reopened and mice were secured on a stereotactic stage with automated thermal support using a Rodent Warmer X1 (Cat #53800M; Stoelting, Wood Dale, IL, USA). A 33 gauge internal cannula (Cat #8IC315IS5SPC; P1 Technologies, Roanoke, VA, USA), with a 4 mm projection below the pedestal, was inserted into a 26 gauge guide cannula (Cat #8IC315GS5SPC, P1 Technologies), with a 3.5 mm projection below the pedestal, and both were connected to PE tubing and secured with a single connector assembly (# C313C/SPC; P1 Technologies). The whole unit was secured vertically with a cannula holder (Cat #505254; World Precision Instruments, Sarasota, FL, USA) and connected to a 22 gauge (51 mm, point style AS) syringe (Cat #80400; Hamilton Company) placed in a Legato 130 syringe pump (Cat #788130; KD Scientific, Holliston, MA, USA). Vehicle (PBS) and drug (GB-13 at concentrations of 50 μg/mL (1 μg dose), 15 μg/mL (0.3 μg dose), or 5 μg/mL (0.1 μg dose)) solutions were subsequently primed through the internal cannula and associated tubing. The cannula holder with attached internal cannula was lowered until flush with the mouse skull to reach the desired injection depth of 4 mm (GBM6) or 4.2 mm (PED17 and SU-DIPGXIII). In all study groups, the same ramped CED infusion protocol was performed with a total volume infused of 20 μL and rates of infusion as follows: 3 μL at 0.2 μL/min, 5 μL at 0.5 μL/min, and 12 μL at 0.8 μL/min [51]. To avoid reflux into the injection tract, the cannula was removed 10 min after completion of infusion. Animals were monitored daily and euthanized at indication of progressive neurologic deficit or if found in a moribund condition.

### 2.8. Immunohistochemistry

Following animal euthanasia by carbon dioxide inhalation, brains were harvested and fixed in 4% paraformaldehyde at room temperature overnight. The brains were then embedded in paraffin and sectioned in the coronal plane (5 μm/section) using a microtome (CM1860 UV; Leica Biosystems, Buffalo Grove, IL, USA). Hematoxylin and eosin (H&E) staining was performed according to standard procedures. For immunohistochemistry (IHC), paraffin-embedded tissue sections were dewaxed in xylene and rehydrated in ethanol. Antigen retrieval was performed by steaming slides in preheated sodium citrate buffer (10 mM tri-sodium citrate, 0.05% Tween 20, pH 6.0) for 30 min. Sections were cooled to room temperature and rinsed with dH20 for 1 min. This was followed by soaking sections in 0.6% hydrogen peroxide in MeOH for 20 min. Sections were then blocked with 10% normal goat serum (NGS) in Tris-buffered saline (TBS) for 30 min at room temperature. Primary antibodies were diluted in TBS with 2% NGS and 0.5% Triton X-100 and applied to sections overnight at 4 °C. Dilution buffer was used instead of primary antibody for tissue-specific negative controls. The next day, sections were washed 3 times for 5 min in TBS with 2% NGS and 0.5% Triton X-100. The VECTASTAIN Elite ABC kit (Cat # PK-6100; VECTOR Laboratory, Burlingame, CA, USA) containing biotinylated secondary antibody was diluted in TBS with 1.5% NGS and added to the sections according to the manufacturer’s recommendations. After 3 additional 5 min washes in TBS, sections were incubated with Avidin/Biotinylated Enzyme Complex (ABC) solution (Cat # PK-6100; VECTOR Laboratory) for 30 min at room temperature. For visualization, the sections were subsequently developed using SignalStain DAB Substrate Kit (Cat # 8059P; Cell Signaling, Danvers, MA, USA) per the manufacturer’s protocol, counterstained with hematoxylin, and mounted with Permount^TM^ (Cat # SP15-100, Thermo Fisher Scientific). Images were acquired with a digital slide scanner (Axio Scan.Z1; Carl Zeiss Microscopy) and are presented at a magnification of 40x. Cell quantification was performed using the Cell Counter plugin for ImageJ (NIH). Ten random fields including a total of 300 to 500 cells were captured for each antibody. Results are presented as the percentage of positive cells versus the entire counted cell population. Low-power images are included to demonstrate consistency of staining in tissue sections. Detailed information regarding antibodies used for immunohistochemistry is provided in Table S3.

### 2.9. Statistical Analysis

The data were collected and presented as the mean ± standard deviation or standard error of the mean when appropriate. Direct statistical comparisons between 2 groups were conducted using two-tailed Student’s *t*-tests. Non-linear least-squares curve fitting was used to determine the potency (IC_50_) of GB-13 treatment in vitro. Survival analysis was performed using the Kaplan–Meier estimate with the Log-Rank test. Statistical tests and analyses were conducted using Prism 9 (GraphPad), with statistical significance set at an α threshold of 0.05, and *p* < 0.05 marked by asterisks in figures.

## 3. Results

### 3.1. IL-13Rα2 Is Expressed at Different Levels in HGG Tumor Cell Models

**Figure 1:**
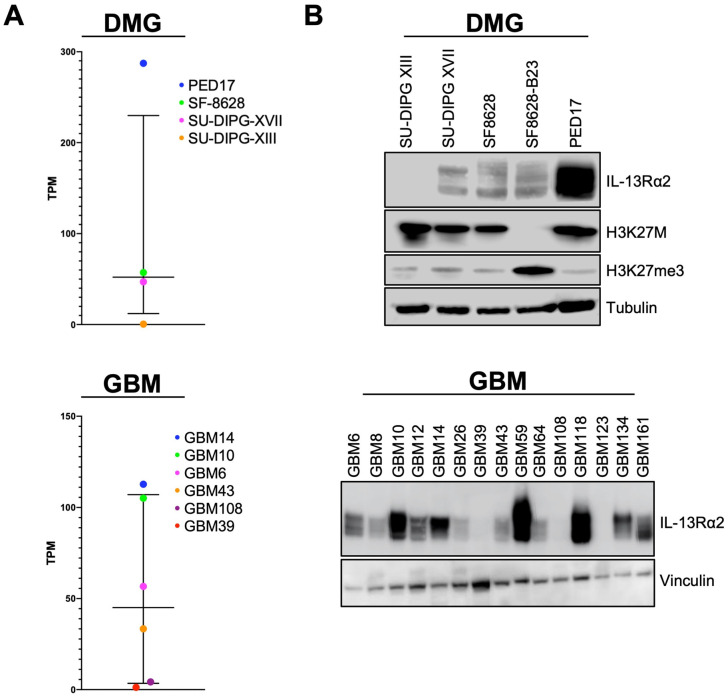
High-grade glioma (HGG) cell lines demonstrate a spectrum of IL-13Rα2 expression on both the mRNA and protein level. (**A**) RNA-Seq and (**B**) immunoblotting of diffuse midline glioma (DMG) and adult glioblastoma (GBM) cell models indicates a correlation between mRNA and protein levels of IL-13Rα2 at baseline. Cell lines differ in terms of IL-13Rα2 expression.

To identify baseline transcript and protein levels of IL-13Rα2 in HGG cells, we performed RNA sequencing and immunoblotting on patient-derived HGG (DMG and adult GBM) cell lines (Figure 1). In accordance with previous investigations [16,52,53], our cohort of sequenced HGG transcriptomes demonstrated highly variable expression of IL-13Rα2 RNA among HGG cell lines (Figure 1A), ranging from low (SU-DIPG XIII-P, GBM39, and GBM108) to intermediate (SU-DIPG XVII, SF8628, GBM43, and GBM6) and high (PED17, GBM10, and GBM14) expression. Next, we evaluated IL-13Rα2 protein levels in available HGG cell lines. IL-13Rα2 protein levels were generally congruent with gene expression in both DMG and adult GBM cell lines (Figure 1B). Several cell models showed high IL-13Rα2 expression, including PED17, GBM10, GBM14, GBM59 and GBM118, while others, such as SU-DIPG XVII, SF8628, SF8628-B23, GBM6, GBM12 and GBM43, showed notably lower (but not absent) IL-13Rα2 levels. IL-13Rα2 expression was not variable between SF8628 and SF8628-B23, indicating expression of this receptor is not impacted by presence of the K27M mutation. A third category of HGG cell lines, including SU-DIPG XIII-P, GBM39, GBM108 and GBM123, demonstrated IL-13Rα2 protein levels that were below the detection threshold of our assay.

### 3.2. Functional Impact of IL-13Rα2 on HGG Proliferation and Survival

**Figure 2:**
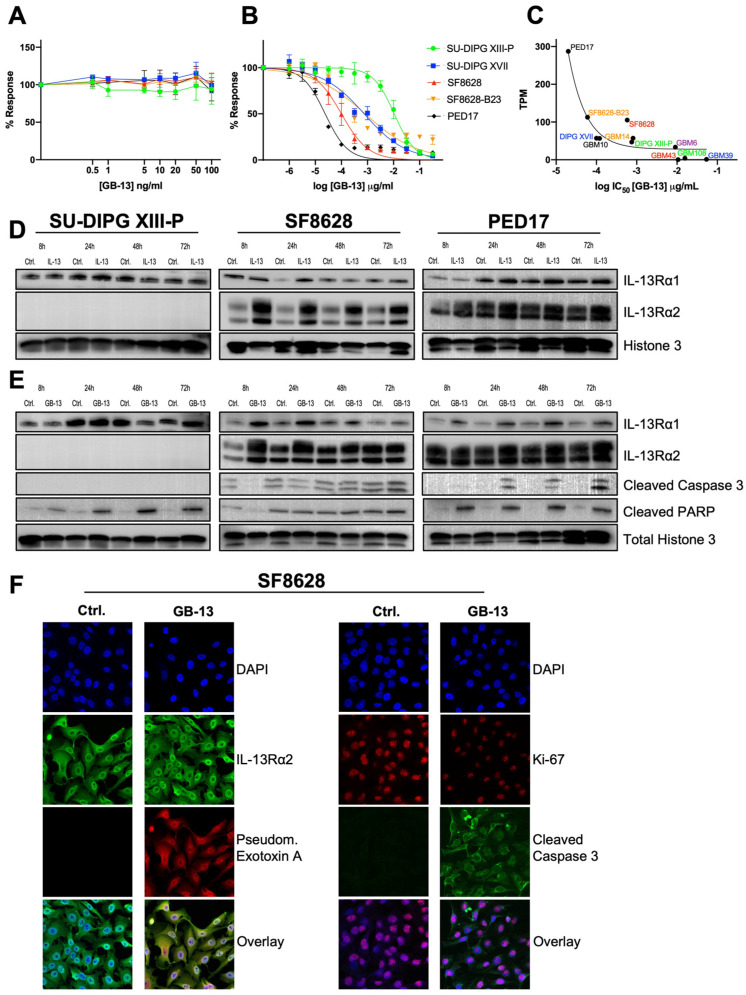
Sensitivity to anti-tumor effects of GB-13 correlates to IL-13Rα2 status and is reflected in apoptosis induction and decreased cell proliferation. (**A**,**B**) Cell proliferation and viability assay of indicated DMG cell lines at escalating doses of IL-13 and GB-13. IL-13 stimulation does not impact cell proliferation. GB-13 decreases cell viability in a dose and IL-13Rα2 level-dependent manner. IC_50_ values were calculated using non-linear least-squares curve fitting. Each drug was tested in triplicate with three independent experiments (*n* = 9) in each cell line and assayed at 72 h. (**C**) Inverse relationship between IL-13Rα2 expression and sensitivity towards GB-13 demonstrated by non-linear least-squares curve fitting (r^2^ = 0.88). (**D**,**E**) Immunoblotting of indicated DMG cell lines after 8, 24, 48, and 72 h of IL-13 (10 ng/mL) and GB-13 (cell line-specific IC_50_) exposure. IL-13 can increase IL-13Rα2 expression. Similar to IL-13, GB-13 does not lead to IL-13Rα2 downregulation over time but rather upregulates the receptor in select cell lines. (**F**) Immunofluorescence staining of SF8628 cells following 72 h of treatment with GB-13 at IC_50_. The *Pseudomonas* exotoxin A (PE) moiety of GB-13 colocalizes to IL-13Rα2, while receptor levels are maintained over prolonged durations of treatment (left). Cells demonstrate increased levels of apoptotic cell death (cleaved caspase 3) and decreased cell proliferation (Ki-67) after exposure to GB-13 (right). Images are representative of three independent experiments.

Given the cell line-dependent overexpression of IL-13Rα2 in our DMG and adult GBM tumor cell models, we investigated the role of IL-13Rα2 signaling in HGG (Figure 2). To determine whether cytokine stimulation by IL-13 impacts cell proliferation in vitro, HGG cells were treated with varying concentrations of the canonical ligand of IL-13Rα2, IL-13. While the lack of SU-DIPG XIII-P response was consistent with the low expression of IL-13Rα2 in the assayed cell models, none of the IL-13Rα2-medium or IL-13Rα2-high cell lines stimulated with IL-13 demonstrated any significant increase in cell proliferation versus media as the control (Figure 2A). Based on previous reports, demonstrating IL-13Rα2 is implicated in cell survival rather than cell growth and invasion [19,52], we hypothesized that cytokine stimulation would be associated with increased IL-13Rα2 expression to enforce this anti-apoptotic effect. To test this, we stimulated HGG cells with IL-13 (10 ng/mL) and investigated protein levels at various time points (Figure 2D). Stimulation with IL-13 resulted in robust upregulation of IL-13Rα2 in IL-13Rα2-medium and IL-13Rα2-high cell lines after 8, 24, 48, and 72 h. Conversely, IL-13Rα1 levels remained unaffected by IL-13 stimulation in all assayed HGG cell models.

### 3.3. GB-13 Elicits Potent Anti-Tumor Effects in HGG Cell Models

To assess whether IL-13Rα2 expression confers sensitivity to IL-13Rα2-targeted therapy in vitro, we tested the pharmacological response of HGG cells to GB-13. We selected 11 HGG (5 DMG and 6 adult GBM) cell lines and exposed them to varying concentrations of GB-13, with treatments ranging from 0.001 to 320 ng/mL. The results showed a direct relationship between IL-13Rα2 expression and GB-13 sensitivity (Figure 2B and Figure S1). GB-13 demonstrated strong cytotoxicity in IL-13Rα2-high cell lines versus comparatively insensitive IL-13Rα2-low cell models (Table 1). The IC_50_ values of GB-13 in IL-13Rα2-high cells were: 0.02 ng/mL for PED17 cells, 0.06 ng/mL for GBM14, and 0.58 ng/mL for GBM10. IL-13Rα2-medium cells displayed the following IC_50_ values for GB-13: 0.10 ng/mL for SF8628, 0.75 ng/mL for SU-DIPG XVII, 0.81 ng/mL for SF8628-B23, 0.12 ng/mL for GBM6, 9.08 ng/mL for GBM43. Finally, the IC_50_ values for GB-13 in IL-13Rα2-low cells were: 10.63 ng/mL for SU-DIPG XIII-P, 15.74 ng/mL for GBM108, and 53.82 ng/mL for GBM39. DMG and adult GBM cell models showed similar sensitivity towards GB-13 dependent on IL-13Rα2 status. High and medium expressors were similarly sensitive (*p* = 0.43) and together had significantly different IC_50_ values compared to IL-13Rα2-low cell lines (*p* = 0.009) (Figure S2). Non-linear least-squares curve fitting demonstrated an inverse relationship between IL-13Rα2 expression and GB-13 cytotoxic effect (r^2^ = 0.88) (Figure 2C).

To gain insight into the effects of GB-13 on IL-13Rα2, we next treated HGG cells with IC_50_ concentrations of the drug and investigated protein levels at 8, 24, 48, and 72 h (Figure 2E). Similar to IL-13 stimulation, GB-13 did not induce IL-13Rα2 downregulation but rather led to stable or increased protein levels over time. Intriguingly, IL-13Rα1 was upregulated in some IL-13Rα2-medium and IL-13Rα2-high cell models exposed to GB-13. Furthermore, while there was some baseline signaling associated with apoptotic cell death in untreated SF8628 cells, apoptosis induction in the GB-13 condition was generally marked by a time-dependent increase in cleaved caspase 3 and/or cleaved PARP levels. These results were confirmed with confocal microscopy (Figure 2F, Figures S3 and S6), where we found prominent IL-13Rα2 levels at baseline, which were retained in cells treated with GB-13 for up to 72 h. By staining for the PE-domain of GB-13, we confirmed colocalization of the drug to the receptor as well as internalization into the cytoplasm and nucleus. In addition to increased levels of apoptosis, cellular proliferation (Ki-67) was decreased in the presence of GB-13.

### 3.4. Intratumoral Administration of GB-13 Results in Decreased Tumor Burden and Prolonged Survival In Vivo

**Figure 3:**
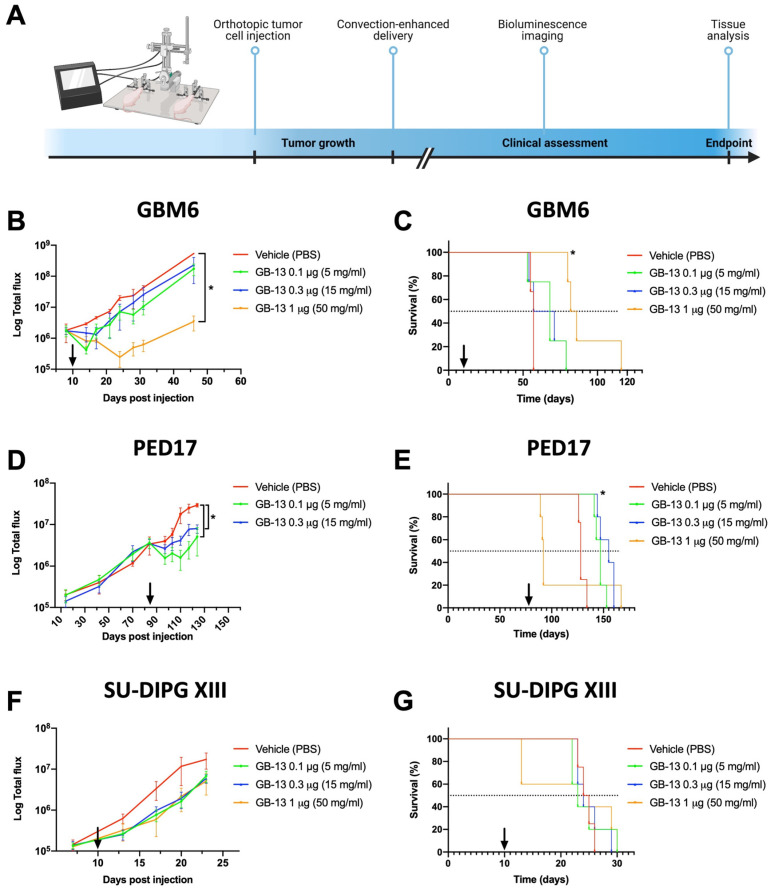
CED infusion of GB-13 results in reduced BLI signals and prolonged survival of IL-13Rα2-upregulated DMG and adult GBM patient-derived xenografts grown as orthotopic tumors. (**A**) Schematic representation of tumor cell injection and CED workflow (created with BioRender.com, accessed on 7 November 2021). Four to five mice were used per treatment group in each cell model (GBM6 is adult GBM with medium IL-13Rα2 levels; PED17 and SU-DIPG XIII-P are DMG with IL-13Rα2-high and IL-13Rα2-low levels, respectively). A single 1 μg dose of GB-13 by CED (arrow) results in decreased (**B**) BLI signals (*p* = 0.01) and (**C**) prolonged survival of GBM6-bearing animals (*p* = 0.01). (**D**) BLI signals in PED17 xenografts are reduced following 0.1 μg (*p* = 0.0001) or 0.3 μg (*p* = 0.0004) of GB-13. (**E**) 0.1 μg (*p* = 0.003) and 0.3 μg (*p* = 0.003) dose levels extend survival without a notable dose–response, but a 1 μg dose of GB-13 is associated with lethal toxicity in 4 out of 5 animals approximately 72 h after the infusion. In SU-DIPG XIII-P animals, CED of GB-13 is not associated with (**F**) reduced BLI signals or (**G**) survival benefit. BLI data are presented as the mean ± standard deviation, and significance between groups was calculated using two-tailed Student’s *t*-tests. Significance of endpoint comparison between treatment groups were calculated using the Log-Rank test.

In order to validate the anti-tumor effects of GB-13 in vivo, we utilized orthotopic patient-derived murine xenograft models of HGG, including IL-13Rα2-low (SU-DIPG XIII-P), IL-13Rα2-medium (GBM6), and IL-13Rα2-high (PED17) models. Tumor-bearing animals were randomized into four cohorts and treated with a single, brain-targeted dose of GB-13 via CED in 4–5 animals per treatment arm (Figure 3A). We initially established our drug delivery system in adult GBM animals by infusing vehicle solution (PBS) or various doses of GB-13 into the hemispheric GBM6 tumor region. All CED systems were placed and tolerated without complications, as previously published [54]. There were no procedure-related deaths, and clinical assessments of animals after completed infusions were all unremarkable with no signs of acute or delayed toxicities or neurological deficits. The tumor volume, measured by BLI, was significantly lower in animals treated with 1 μg of GB-13 (*p* = 0.01) as compared to the 0.3 μg- (*p* = 0.14), 0.1 μg- (*p* = 0.08) and vehicle-treated groups (Figure 3B). A single dose of 1 μg GB-13 significantly prolonged survival, with a median survival of 84 days (*p* = 0.01) in comparison to 64 days in 0.3 μg GB-13 (*p* = 0.35), 68 days in 0.1 μg GB-13 (*p* = 0.17) and 57 days in vehicle groups (Figure 3C).

**Figure 4:**
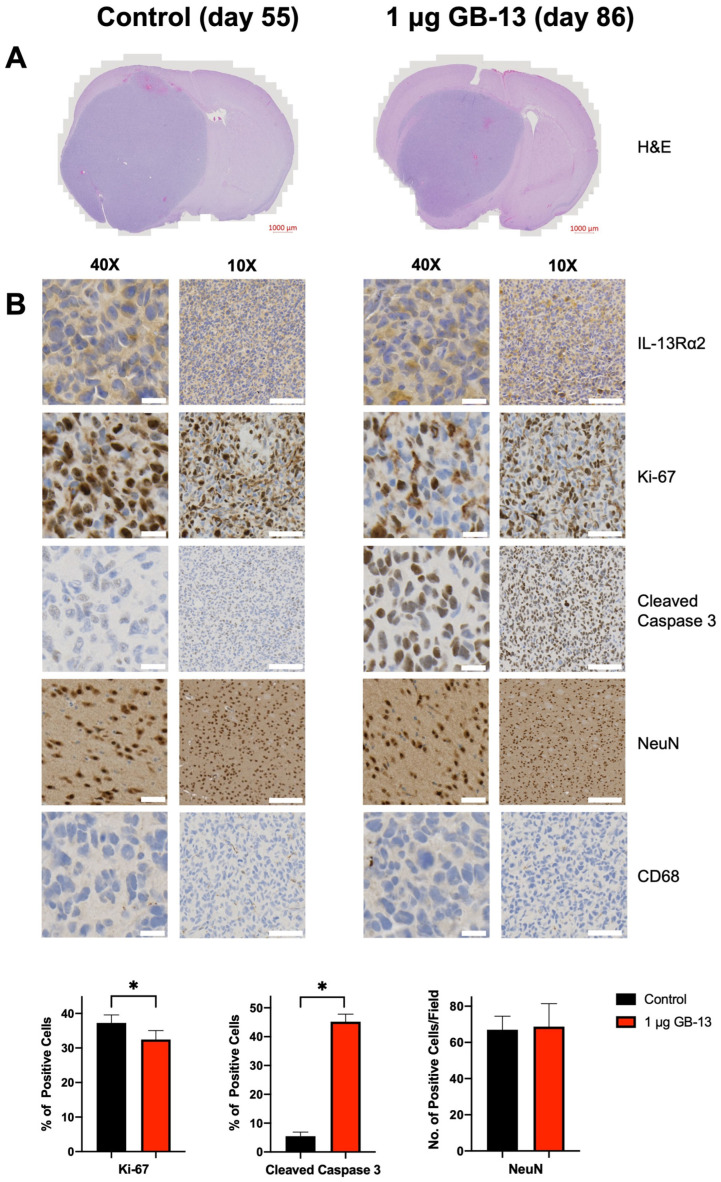
Immunohistochemistry of HGG-bearing mouse brains harvested when moribund. Presented samples were harvested on days 55 and 86 following CED of vehicle solution or GB-13 at a dose of 1 μg, respectively. Images are representative of four mice in each group. (**A**) Corresponding H&E demonstrates maintained tissue architecture and decreased tumor size after GB-13 treatment. (**B**) While IL-13Rα2 status is retained after a single CED infusion of GB-13, treatment leads to decreased cell proliferation (*p* = 0.03) and increases the number of apoptotic cells (*p* < 0.0001). The density of NeuN-positive neuronal (NeuN+) cells is retained in ipsilateral brain regions (*p* = 0.82). CD68+ monocyte infiltrate is not evidenced following GB-13 exposure. Scale bars: 40×: 20 μm, 10×: 100 μm.

Histologic evaluation of brains from mice euthanized in a moribund state demonstrated maintained tissue architecture and decreased tumor size after GB-13 treatment (Figure 4A). On-target drug effects were validated in tumors by IHC analysis of IL-13Rα2 levels, apoptosis induction, and cellular proliferation in drug-treated mice compared to control (Figure 4B and Figure S6). In agreement with the in vitro data, high IL-13Rα2 status was retained in GBM6 cells. Cellular proliferation, which was determined by Ki-67 staining, was decreased following exposure to GB-13 (*p* = 0.03). Intriguingly, intense staining for the apoptosis marker cleaved caspase 3 was evidenced throughout the tumor area in all GB-13 groups but largely absent in vehicle-treated animals weeks after GB-13 administration (*p* < 0.0001). To address toxicity considerations that may accompany immunotoxin delivery into the brain, we performed additional IHC analyses for NeuN, a marker of mature neurons, and CD68, which is expressed in high levels by microglia and monocytes. CED of GB-13 did not result in a decrease in NeuN-positive (NeuN+) cells in the infused, ipsilateral hemisphere as compared to vehicle (*p* = 0.82). No CD68+ immune cell infiltration was evidenced in any study group.

We next sought to validate these findings in an IL-13Rα2-upregulated DMG xenograft model. PED17 cells were orthotopically implanted into the pons, and tumor-bearing animals were again treated with vehicle solution, 0.1, 0.3, or 1 μg of GB-13. In line with prior observations, all animals tolerated the CED procedure; however, at the highest dose (a 1 μg CED infusion of GB-13), marked signs of toxicity developed in all five animals within 24 h of infusion (neurological deficits such as hemiparesis or ataxia, hunched body position, dermatitis), and 4 of 5 animals had to be euthanized within 72 h of drug administration. Post-operative clinical assessments were unremarkable for animals treated with 0.1 or 0.3 μg of GB-13. Comparison of BLI signal demonstrated that a single 0.1 or 0.3 μg GB-13 infusion significantly decreased tumor volume (*p* = 0.0001 and 0.0004, respectively; Figure 3D) and significantly extended median survival (147 days for 0.1 μg GB-13 (*p* = 0.003) and 155 days for 0.3 μg GB-13 (*p* = 0.003)) compared to the vehicle group (128 days) (Figure 3E). Similar to the findings in hemispheric GBM6 tumors, none of the 0.1 or 0.3 μg doses had an impact on NeuN+ cell density and CD68+ cell infiltration. Consistent with the observed differential in clinical toxicity, a 1 μg dose of GB-13 resulted in a marked decrease in NeuN-positive cells in the brainstem as compared to lower GB-13 doses or vehicle control (*p* = 0.02). There was no evidence of CD68+ monocyte cell infiltration following exposure to 1 μg GB-13. Detected levels of IL-13Rα2 remained constant among treatment groups. A decrease in DMG-characteristic H3 K27M and increase in H3 K27me3 was evidenced in drug-treated tumors. Additional IHC findings were similar to the results of the first study (Figures S4 and S6).

Finally, we used the DMG cell line SU-DIPG XIII-P to establish a brainstem HGG xenograft model with low IL-13Rα2 protein levels. Based on the in vitro data, we did not expect GB-13 to impact tumor volume or survival using the previously established dosing regimen. Indeed, CED of 0.1, 0.3, or 1 μg of GB-13 failed to demonstrate significant tumor growth reduction (*p* = 0.16, 0.18 and 0.27, respectively; Figure 3F) and did not provide profound survival benefit compared to control animals (24.5 days in vehicle, 23 days in 0.1 μg GB-13 (*p* = 0.92), 24 days in 0.3 μg GB-13 (*p* = 0.68) and 24 days in 1 μg GB-13 (*p* = 0.57)) (Figure 3G). While the CED procedure proved to be feasible and safe among all treatment groups, 2 of 5 animals dosed with 1 μg of GB-13 had to be euthanized within 72 h of drug administration. No CD68+ immune cell infiltration or reduction in NeuN-positive cells was observed in mice treated with 0.1 or 0.3 μg of GB-13, there was again evidence of reduced NeuN+ cell density in the brainstem of animals treated with 1 μg of GB-13. IHC did not show increased staining for cleaved caspase 3 in GB-13 drug-treated IL-13Rα2-low xenografts, and a high degree of cellular proliferation was retained in these tumors after GB-13 infusion. In accordance with in vitro protein-level analysis, IHC staining for IL-13Rα2 was absent in SU-DIPG XIII-P xenografts. Furthermore, H3 K27M and H3 K27me3 remained largely unchanged in vehicle versus GB-13-treated tumors (Figures S5 and S6). These results suggest that moderate to high IL-13Rα2 expression is required for targeted therapies such as GB-13 to impart therapeutic effect in HGG orthotopic xenograft models.

## 4. Discussion

To date, the search for effective treatment against HGGs has not substantially improved outcomes [55,56,57,58]. IL-13Rα2 represents a clinically validated target in HGG therapy [33,34,36]. In this study, we identified a favorable correlation between IL-13Rα2 mRNA and protein levels. IL-13Rα2 status predicted the efficacy of a novel IL-13 immunotoxin, GB-13, in cultured HGG cells. Intraparenchymal administration of GB-13 into orthotopically implanted patient-derived xenograft models of HGG was feasible, and a single therapeutic infusion of GB-13 using CED significantly ameliorated tumor burden and resulted in significant prolongation in survival of animals harboring IL-13Rα2-upregulated orthotopic xenografts, underscoring the promise of IL-13Rα2 targeting in the context of HGG with increased IL-13Rα2 expression.

IL-13Rα2 has long been recognized as a prognostic biomarker for poor disease prognosis in brain tumors including HGG [16,32,59]. While the negligible impact of canonical ligand-mediated IL-13Rα2 stimulation on cell proliferation in IL-13Rα2-high versus IL-13Rα2-low cell lines was somewhat surprising, similar results have been previously reported in cell growth and invasion studies [52]. Recent investigations have elucidated the role of IL-13Rα2 as a tumor-associated antigen that mediates aberrant STAT3 signaling, driving increased expression of anti-apoptotic genes and, ultimately, promoting tumor progression by blocking cell death and mediating survival [17,59,60]. These findings mirror those observed in this study, in that IL-13Rα2-upregulated HGG cells selectively increase expression of IL-13Rα2, but not IL-13Rα1, the functional counterpart, when exposed to IL-13, a natural IL-13Rα2-binding partner. We were able to expand these and previous observations by demonstrating that binding of GB-13 equally induces upregulation of IL-13Rα2 in IL-13Rα2-expressing HGG cells [61,62]. Together, this holds promise for IL-13Rα2-targeting agents to achieve repeated and durable response in HGG with increased IL-13Rα2 levels.

Previous efforts to target IL-13Rα2 by various treatment modalities suggest the utility of cellular immunotherapy or immunoconjugates for HGG therapy [32,33,63,64]. The clinical effect of immunotoxins is heavily linked to payload efficacy. Consequently, a pivotal requirement in the design of an immunotoxin is the selection of an exceedingly potent cytotoxic capable of inducing cellular death at low (~10 nM) concentrations [65]. The foundational efforts of Puri and colleagues in developing cintredekin besudotox have demonstrated the feasibility of creating such a molecule for killing of IL-13Rα2-expressing cells by linking a truncated form of PE to human IL-13 [66]. GB-13 is essentially the successor of cintredekin besudotox, featuring refinements to both the targeting moiety and the payload domain [24]. We observed favorable potency with GB-13 compared to previous reports of cintredekin besudotox in IL-13Rα2-upregulated HGG cell lines [42,66,67,68,69]. Interestingly, the cytotoxic effect of GB-13 was highly dependent on IL-13Rα2 status, with GB-13 demonstrating dose-depending killing at concentrations more than 100-fold lower in IL-13Rα2-high versus IL-13Rα2-low HGG cells. This observation, whereas potentially underappreciated by previous in vitro investigations of IL-13Rα2-targeting agents, has profound implications for the clinical application of these therapeutics in HGG, where IL-13Rα2 overexpression is detected in a subset of, but not all, tumors [15,23,30,31].

Delivery of immunotoxins via CED is a valuable approach to circumvent the BBB and ensure brain-targeted drug delivery of cytotoxic payloads; however, there is growing evidence that this strategy has unique pitfalls to consider. A phase I study using single-catheter convective infusion of cintredekin besudotox in pediatric patients with DIPG and supratentorial HGG was terminated because the drug did not to reach the predefined distribution volume to cover the entire tumor area on MRI and MR spectroscopy [34]. While several phase I/II studies of CED with cintredekin besudotox for adult patients with HGG showed promise [33,36,64], the only phase III clinical trial, the PRECISE study, failed to demonstrate a 50% improvement in median survival over Gliadel wafers [33]. Extensive post-trial analyses investigated possible explanations for the lack of efficacy observed. While technical issues surrounding catheter placement and drug distribution are surmountable in future studies by optimizing catheter design and positioning of potentially multiple catheters, there was limited consideration of target expression for patient inclusion [37,70]. A large scale analysis of clinical trials utilizing biomarkers found significant improvement in trial success relative to no biomarker inclusion criteria [71]. Consistent with this idea, we compared IL-13Rα2 mRNA and protein levels from homogeneous cell populations, which showed that gene expression correlates with IL-13Rα2 protein status. The herein presented findings in HGG xenograft models with low, medium and high IL-13Rα2 levels demonstrate that GB-13 decreases tumor volume and prolongs survival in a manner strongly associated with IL-13Rα2 status. Although no benefit was observed in animals harboring IL-13Rα2-low HGG, IL-13Rα2-medium and IL-13Rα2-high animals had significantly reduced tumor burden and lived significantly longer than vehicle-treated animals, indicating IL-13Rα2 may not only be a therapeutic target but also a predictive biomarker for future clinical trial patient inclusion.

The moderate anti-tumor efficacy of GB-13 across multiple IL-13Rα2-expressing HGG models provides some support for an early-phase clinical trial in patients with upregulated IL-13Rα2 expressing tumors. However, our study also highlight several limitations. First, high-dose CED infusion into the brainstem of HGG-bearing mice was associated with profound toxicity, which indicates a narrow therapeutic window for this agent when delivered directly into eloquent brain regions. This is especially notable since a previous in vivo study of intra-tumoral GB-13 in a murine model of malignant peripheral nerve sheath tumor did not find dose-limiting toxicities [24]. Furthermore, observed toxicities in a phase I clinical trial of cintredekin besudotox delivered by CED into the brainstem of DIPG patients were limited to transient cranial nerve deficits and lethargy after infusion [72]. While the high local concentrations of infused GB-13 in the comparatively small mouse brainstem likely contributed to the observed peritumoral toxicity in our study, the data presented warrant further investigation before moving into clinical testing. Second, our findings are limited to a single therapeutic infusion of GB-13. Previous studies using CED to deliver small molecules to the brain have shown that these drugs were rapidly cleared from the infusion site [73]. Although large biomolecules such as GB-13 are likely to remain in the brain for longer periods of time, clinical applications may require multiple infusions or longer infusion durations to achieve adequate drug distribution and sustained therapeutic effect. To this extent, a recent clinical trial has demonstrated safety of sequential CED infusions into the pediatric brainstem [74]. Finally, we evaluated GB-13 as a monotherapy, which has obvious limitations considering the long history of negative clinical trials for HGG. The efficacy of IL-13Rα2-targeted therapy has previously been shown to be enhanced by both radiation and cytotoxic chemotherapy [36,75]. Efficacious in HGG with high IL-13Rα2 levels, future studies should investigate GB-13 as part of a comprehensive treatment regimen, including a rational combination of therapeutic strategies that have previously demonstrated to be beneficial in this devastating disease.

## 5. Conclusions

HGGs encompass a large proportion of malignant tumors within the central nervous system. Up to 80% of HGG overexpress the tumor-associated receptor IL-13Rα2. Despite advances in our understanding of underlying disease mechanisms, the prognosis for HGG remains dismal and efficacious therapies are lacking. As such, there is a dire, unmet, gap in clinical practice for treating this devastating disease. Here, we investigated the pharmacological effects of GB-13, a novel tumor-specific immunotoxin that contains an engineered mutant of human IL-13 fused to a cytotoxic PE molecule. Administration of GB-13 demonstrated a promising pharmacological response in DMG and adult GBM models both in vitro and in vivo in a manner strongly associated with IL-13Rα2 status, underscoring the potential of this IL-13Rα2-targeted therapy in a subset of HGG with increased IL-13Rα2 expression.

## Figures and Tables

**Table 1:** Fifty % inhibitory concentration (IC_50_) values of GB-13.

Cell Line	IC_50_ Value (ng/mL)
SU-DIPG XIII-P	10.63
SU-DIPG XVII	0.75
SF8628	0.10
SF8628-B23	0.81
PED17	0.02
GBM6	0.12
GBM10	0.58
GBM14	0.06
GBM39	53.82
GBM43	9.08
GBM108	15.74

## Data Availability

All data supporting reported results can be found in the manuscript and Supplementary Materials to the manuscript.

## Supplementary Materials

The following supporting information can be downloaded at: https://www.mdpi.com/article/10.3390/pharmaceutics14050922/s1, Figure S1: Cell viability and dose–response curve of adult GBM cell models; Figure S2: DMG and adult GBM cell lines demonstrate similar sensitivity towards GB-13 dependent on IL-13Rα2 status; Figure S3: Immunofluorescence of SF8628 cells, a DMG cell line, after 8, 24, 48, and 72 h of GB-13 exposure at IC_50_; Figure S4: Immunohistochemistry of PED17-bearing mouse brains harvested at endpoint; Figure S5: Immunohistochemistry of SU-DIPG XIII-P-bearing mouse brains harvested at endpoint; Figure S6: Negative staining controls for immunofluorescence and immunohistochemistry experiments; Table S1: Details regarding the cell lines used in this study; Table S2: Media composition for culture of patient-derived cell lines; Table S3: Antibodies used for immunoblotting, immunofluorescence and immunohistochemistry.
