# Supplementary material for: IL-13Rα2 Status Predicts GB-13 (IL13.E13K-PE4E) Efficacy in High-Grade Glioma
Source: Pharmaceutics. 2022 Apr 24;14(5):922. doi: 10.3390/pharmaceutics14050922 (PMC9143740; doi:10.3390/pharmaceutics14050922)
Supplement: Supplementary file 1 [file pharmaceutics-14-00922-s001.zip › pharmaceutics-1689426-supplementary.pdf]

Supplementary Materials: IL-13Rα2 Status Predicts GB-13 (IL13.E13K-PE4E) Efficacy in High-Grade Glioma

Julian S. Rechberger, Kendra A. Porath, Liang Zhang, Cody L. Nesvick, Randy S. Schrecengost, Jann N. Sarkaria and David J. Daniels

Table S1. Details regarding the cell lines used in this study.

Cell line ID	Age/Sex	Institution	Molecular Status	Tests	Date of Tissue Collection	Culture Media
GBM6	65/M	Mayo Clinic	GBM, IDH WT	WES, Sanger, STR	2000	FBS or Stem
GBM8	75/F	Mayo Clinic	GBM, IDH WT	WES, Sanger, STR	2000	FBS or Stem
GBM10	40/M	Mayo Clinic	GBM, IDH WT	WES, Sanger, STR	2001	FBS or Stem
GBM12	69/M	Mayo Clinic	GBM, IDH WT	WES, Sanger, STR	2001	Stem
GBM14	58/M	Mayo Clinic	GBM, IDH WT	WES, Sanger, STR	2001	FBS or Stem
GBM26	49/M	Mayo Clinic	GBM, IDH WT	WES, Sanger, STR	2002	FBS
GBM39	51/M	Mayo Clinic	GBM, IDH WT	WES, Sanger, STR	2003	FBS or Stem
GBM43	69/M	Mayo Clinic	GBM, IDH WT	WES, Sanger, STR	2003	FBS or Stem
GBM59	83/F	Mayo Clinic	GBM, IDH WT	WES, Sanger, STR	2004	FBS
GBM64	63/M	Mayo Clinic	GBM, IDH WT	WES, Sanger, STR	2006	FBS or Stem
GBM108	62/M	Mayo Clinic	GBM, IDH WT	WES, Sanger, STR	2009	FBS or Stem
GBM118	56/F	Mayo Clinic	GBM, IDH WT	WES, Sanger, STR	2009	FBS
GBM123	62/F	Mayo Clinic	GBM, IDH WT	WES, Sanger, STR	2009	FBS or Stem
GBM134	42/F	Mayo Clinic	GBM, IDH WT	WES, Sanger, STR	2009	FBS
GBM161	78/M	Mayo Clinic	Glioscarcoma, IDH WT	WES, Sanger, STR	2012	FBS
PED17	12/F	Mayo Clinic	DMG, H3.3 K27M	Sanger, STR	2015	MHM
SU-DIPG XIII-P	6/F	Stanford University	DMG, H3.3 K27M	Sanger, STR	Received 2016	TSM
SU-DIPG XVII	8/M	Stanford University	DMG, H3.3 K27M	Sanger, STR	Received 2016	MHM
SF8628	3/F	Millipore	DMG, H3.3 K27M	Sanger, STR	Purchased 2019	dBT
SF8628-B23	–	Hormel Institute	DMG, H3.3 K27M KO	Sanger, STR	Created 2019	dBT

Abbreviations: GBM = glioblastoma, IDH = isocitrate dehydrogenase, DMG = diffuse midline glioma, WES = whole exome sequencing, STR = short tandem repeat, WT = wildtype.

Table S2. Media composition for culture of patient-derived cell lines.

Cell Lines	Media Name	Media Contents
GBM6, GBM8, GBM10, GBM14, GBM26, GBM39, GM43, GBM59, GBM64, GBM108, GBM118, GBM123, GBM134, GBM161	FBS-containing medium (FBS)	DMEM (Thermo Fisher), 10% FBS (Atlanta Biolocials), and 1% penicillin/streptomycin (Thermo Fisher).
GBM6, GBM8, GBM10,GBM12, GBM14, GBM39, GM43, GBM64, GBM108, GBM123	Stem cell medium (Stem)	KnockOut DMEM/F12 (Thermo Fisher), StemPro NSC SFM supplement (Thermo Fisher), 20ng/mL FGF basic recombinant human (Thermo Fisher), 20ng/mL EGF recombinant human(Thermo Fisher), 200mM L-glutamine (Corning), and 1% penicillin/streptomycin (Thermo Fisher).
PED17, SU-DIPG XVII	Serum-free complete medium (MHM)	DMEM/F12 (HyClone), 25 mM glucose (Sigma-Aldrich), 8.9 mM sodium bicarbonate (Life Technologies), 2 mM glutamine (Life Technologies), 4 mM HEPES (Life Technologies), 1% penicillin/streptomycin (Life Technologies), N2 supplement (Life Technologies), 4 μg/mL heparin (Sigma Aldrich), 20 ng/mL human EGF (PeproTech US), 20 ng/mL human b-FGF (PeproTech US), and 20 ng/mL human PDGF AA with 20 ng/mL human PDGF BB (Shenandoah Biotechnology).
SU-DIPG XIII	Tumor stem cell medium (TSM)	Neurobasal(-A) (Life Technologies), DMEM/F12 (Life Technologies) B27(-A) (Life Technologies), 4 μg/mL heparin (Sigma Aldrich), 20 ng/mL human EGF (PeproTech US), 20 ng/mL human b-FGF (PeproTech US), 20 ng/mL human PDGF AA with 20 ng/mL human PDGF BB (Shenandoah Biotechnology), and 1% penicillin/streptomycin (Life Technologies).
SF8628, SF8628-B23	dBT medium	DMEM/F12 (HyClone), 10% FBS, and 1% penicillin/streptomycin (Life Technologies).

Table S3. Antibodies used for immunoblotting, immunofluorescence and immunohistochemistry.

Antibodies	Source	Identifier	Dilution for WB	Dilution for IF	Dilution for IHC
Cleaved PARP	Cell Signaling	5625S	1:1000		
Cleaved Caspase 3	Cell Signaling	9664S	1:1000	1:500	1:200
IL-13Rα1	Abcam	Ab79277	1:1000		
IL-13Rα2	Cell Signaling	85677	1:1000	1:200	
H3K27M	Abcam	190631	1:3000		1:500
H3K27me3	Cell Signaling	9733S	1:1000		1:100
Histone H3	Cell Signaling	3638S	1:1000		
α-Tubulin	Sigma	T9026	1:20,000		
Vinculin	Cell Signaling	13901S	1:1000		
Pseudomonas Exotoxin A	Sigma	P2318		1:500	
Ki-67	Invitrogen	14-5698-82		1:200	
IL-13Rα2	R&D	AF146			1:250
Ki-67	Dako	GA626			1:400
NeuN	Sigma	MAB377			1:2400
CD68	Abcam	Ab125212			1:800

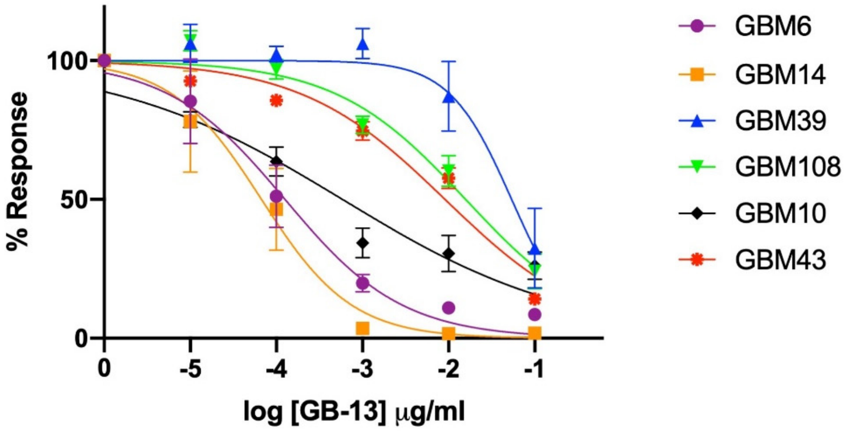

Figure S1. Cell viability and dose-response curve of adult GBM cell models. Similar to other HGG cell lines tested, GB-13 treatment of adult GBM cell lines elicits potent anti-tumor responses in an IL-13Rα2-dependent fashion. IC_{50} values were determined using nonlinear least-squares curve-fitting analysis. GB-13 was tested in triplicate with 3 independent experiments ($n = 9$) in each cell line. Analysis was performed after 72 h of drug exposure.

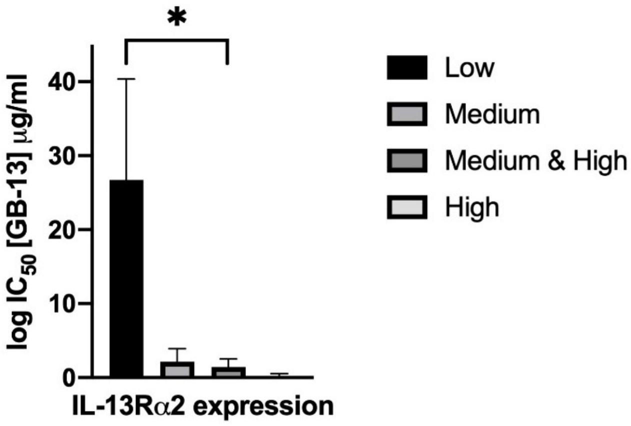

Figure S2. DMG and adult GBM cell lines demonstrate similar sensitivity towards GB-13 dependent on IL-13Rα2 status. High and medium expressors are similarly sensitive ($p = 0.43$) and together have significantly different IC_{50} values compared to IL-13Rα2-low cell models ($p = 0.009$).

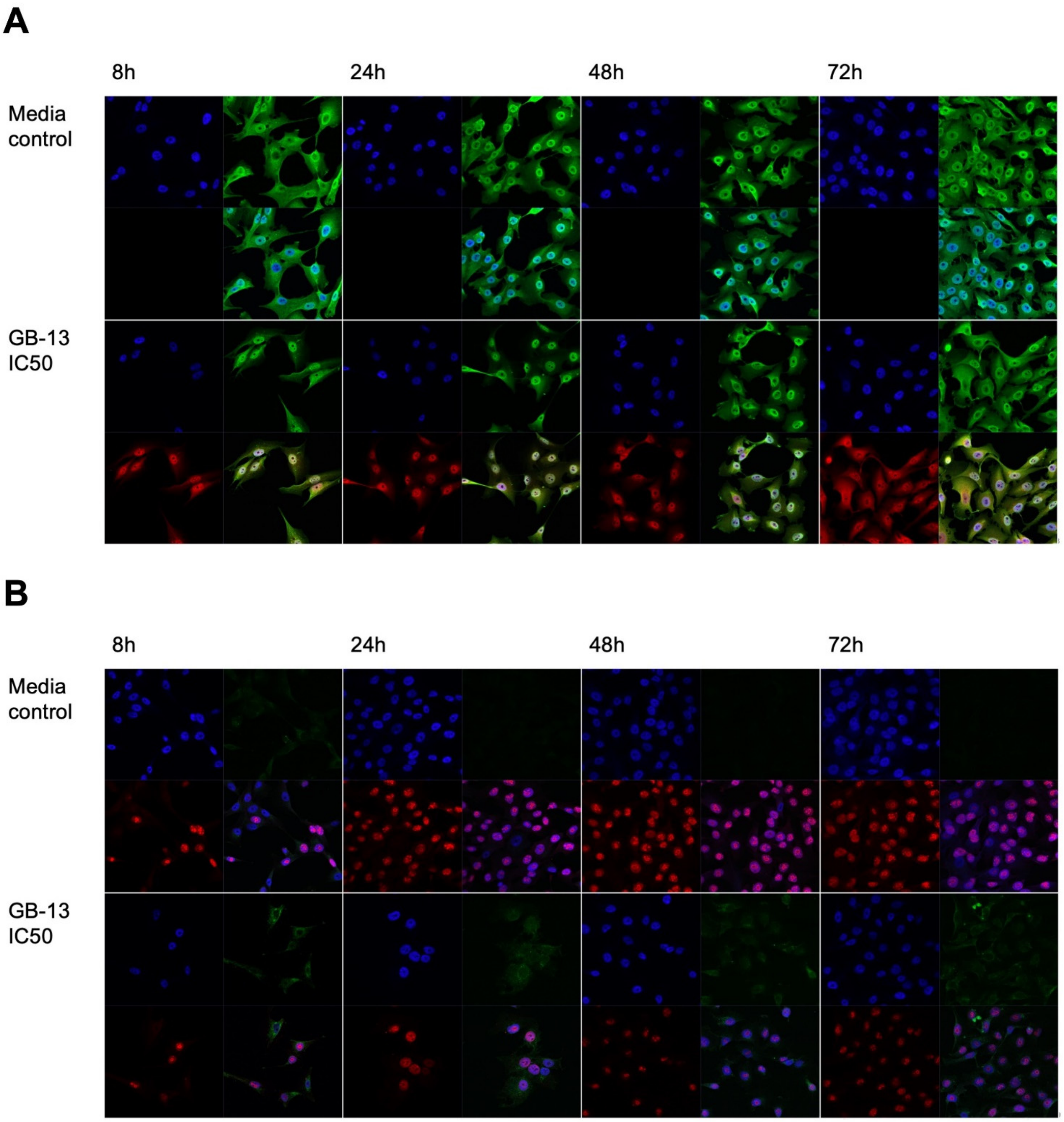

Figure S3. Immunofluorescence (IF) of SF8628 cells, a DMG cell line, after 8, 24, 48, and 72 h of GB-13 exposure at IC_{50}. (**A**) The PE moiety of GB-13 (red) colocalizes to IL-13Rα2 (green) while receptor levels are maintained over prolonged durations of treatment. DAPI shown in blue. Overlay in bottom right of each image. (**B**) Cells demonstrate increased levels of apoptosis (cleaved caspase 3; green) and decreased cell proliferation (Ki-67; red) after exposure to GB-13. DAPI shown in blue. Overlay in bottom right of each image. Images are representative of 3 independent experiments.

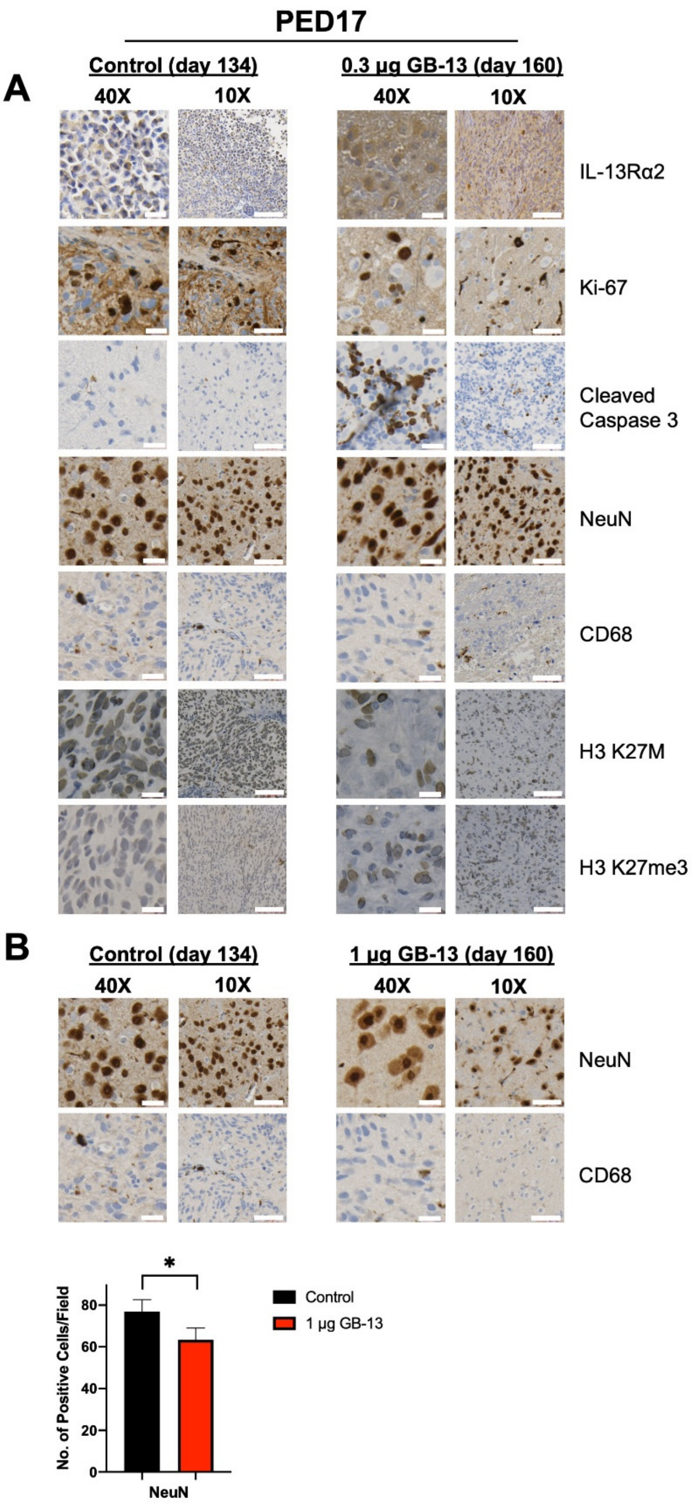

Figure S4. Immunohistochemistry (IHC) of PED17-bearing mouse brains harvested at endpoint. Images are representative of 5 mice in each group. (**A**) Animals were euthanized at day 134 and 160 (treatment with vehicle and GB-13 at a dose of 0.3 mg, respectively). IL-13Rα2 status retained after a single CED infusion of 0.3 mg GB-13. Treatment leads to decreased cell proliferation and increased levels of apoptosis. The density of NeuN+ cells is retained and infiltrative CD68+ monocytes are absent in the infused brainstem. Staining for H3 K27M decreases and H3 K27me3 increases in GB-13 treated tumors. (**B**) A 1 mg CED infusion of GB-13 decreases NeuN+ cell density in the brainstem as compared to lower GB-13 doses or vehicle control. CD68+ monocyte infiltrate is not evidenced following exposure to 1 mg GB-13. Scale bars: 40×: 20 mm, 10×: 100 mm.

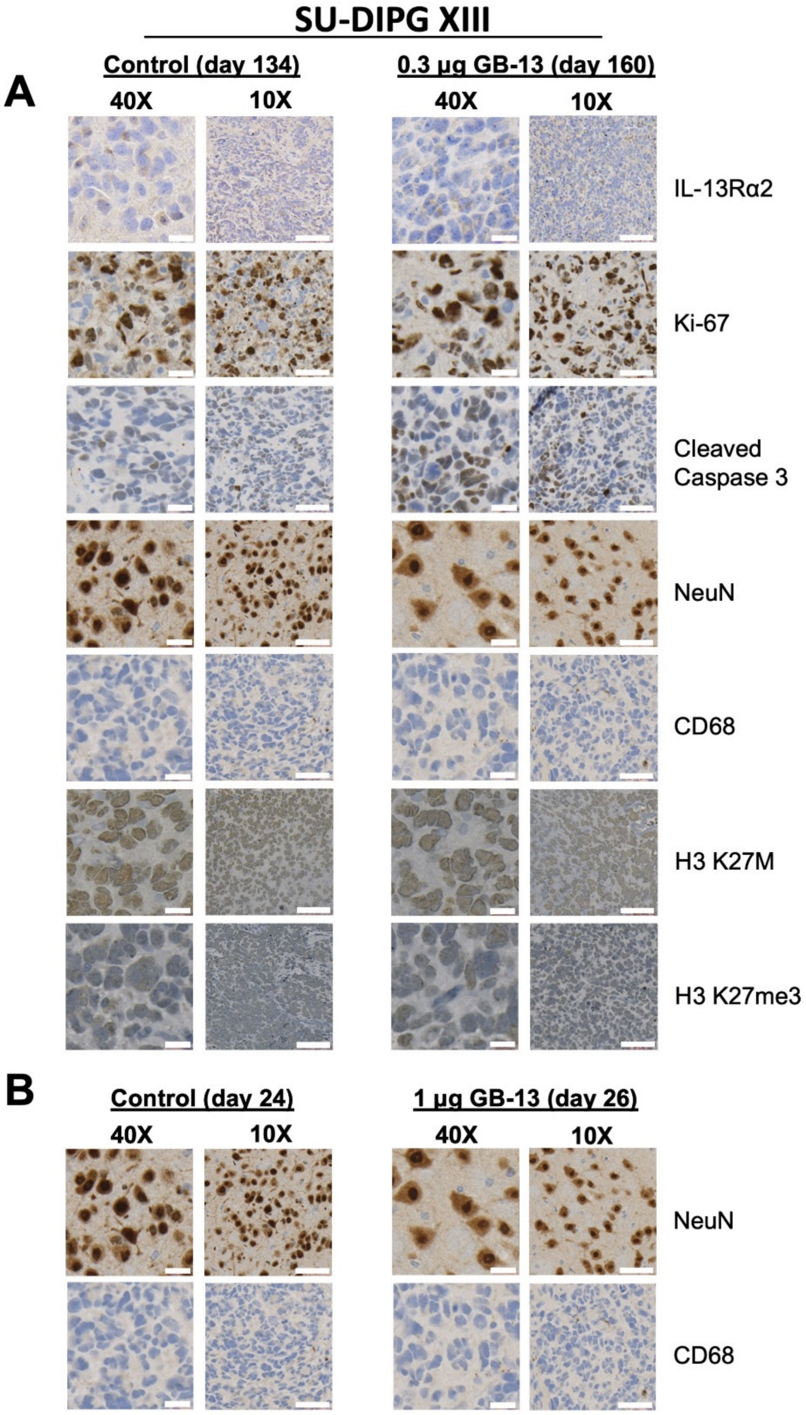

Figure S5. IHC of SU-DIPG XIII-P-bearing mouse brains harvested at endpoint. Images are representative of 5 mice in each group. (**A**) Animals were euthanized at day 24 and 26 (treatment with vehicle and GB-13 at a dose of 0.3 mg, respectively). IL-13Rα2 levels were undetectable after a single CED infusion of vehicle or 0.3 mg GB-13. Treatment did not lead to decreased cell proliferation, and levels of apoptosis were equally low following treatment with vehicle or GB-13. NeuN+ cells density is retained and infiltrative CD68+ monocytes are absent in the infused brainstem. The staining pattern for H3 K27M and H3 K27me3 remains constant following GB-13 treatment. (**B**) A 1 mg CED infusion of GB-13 decreases NeuN+ cell density in the brainstem as compared to lower GB-13 doses or vehicle control. CD68+ monocyte infiltrate is not evidenced following exposure to 1 mg GB-13. Scale bars: 40×: 20 mm, 10×: 100 mm.

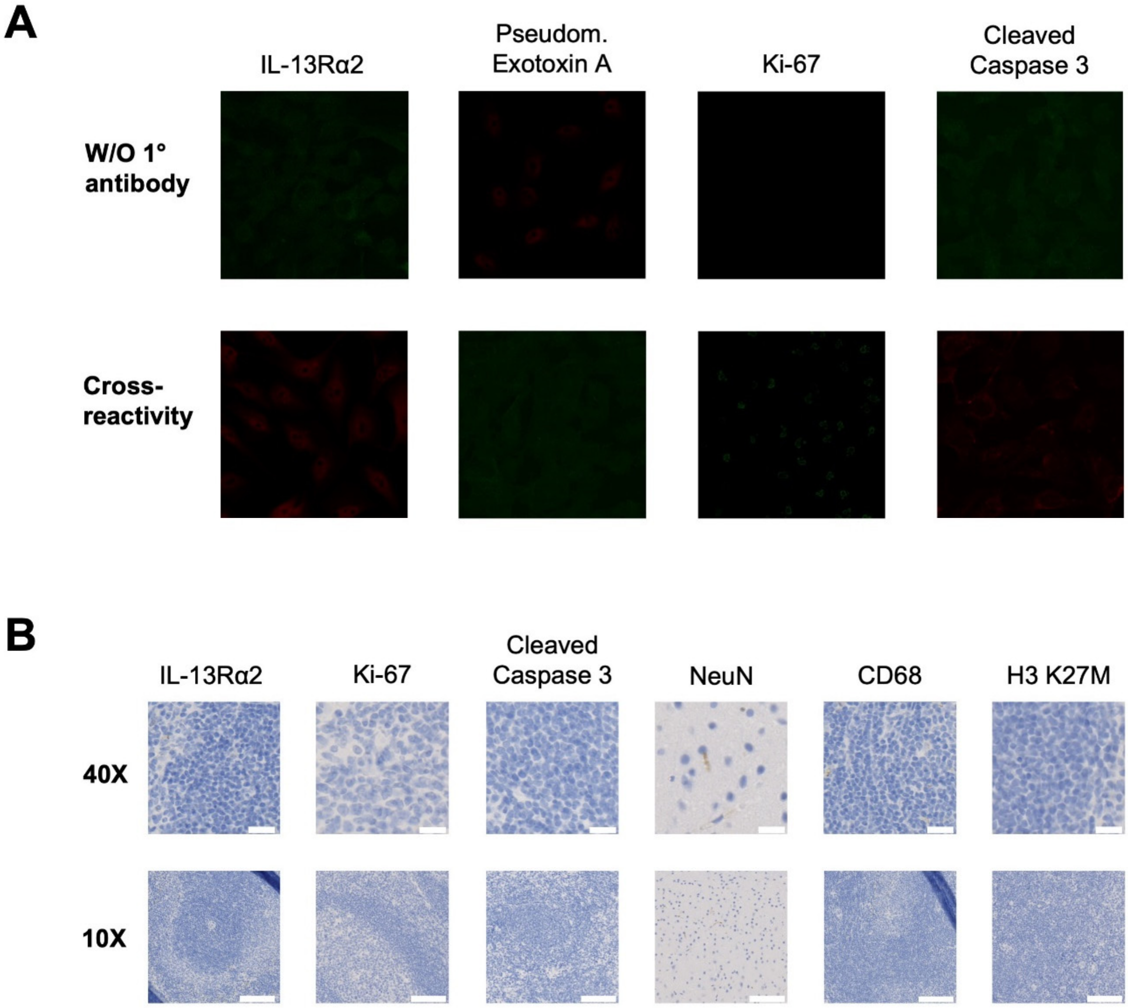

Figure S6. Negative staining controls for IF and IHC experiments. (**A**) A negative control lacking primary antibody was included to confirm the specificity of IF staining (upper panel). Since multi-color IF using two primary antibodies from different hosts was performed, one control per primary antibody condition was included by applying the other secondary antibody to the primary antibody to test for cross-reactivity (lower panel). (**B**) Sections from the following tissues known not to express the target antigen were stained to check for non-specific signal and false positive results in IHC staining: Mouse spleen (IL-13Rα2), normal human tonsil (Ki-67, cleaved caspase 3, H3 K27M), mouse brain (NeuN), and mouse spleen (CD68). Untreated DMG samples served as negative control for H3 K27me3 (see Figures S4 and S5.). Scale bars: 40×: 20 mm, 10×: 100 mm.
